# A Vascular Plug for Persistent Shock Following Transcatheter Mitral Valve Replacement

**DOI:** 10.1016/j.jaccas.2024.102507

**Published:** 2024-09-04

**Authors:** Asad J. Torabi, Purva Patel, Natalia Reborido, Michelle C. Morris, Jeffrey Everett, Anjan Sinha

**Affiliations:** a Division of Cardiovascular Medicine, Indiana University School of Medicine, Indianapolis, Indiana, USA; b Division of Internal Medicine, Indiana University School of Medicine, Indianapolis, Indiana, USA; c Division of Cardiovascular Surgery, Indiana University School of Medicine, Indianapolis, Indiana, USA

**Keywords:** cardiac tamponade, left ventricular perforation, transcatheter mitral valve replacement

## Abstract

A 78-year-old woman with severe bioprosthetic mitral valve degeneration underwent successful transcatheter mitral valve replacement with a valve-in-valve procedure. This case postprocedure was complicated by cardiogenic shock from left ventricular perforation and underscores the importance of the accurate assessment and treatment of patients following transcatheter valvular procedures.

## Graphical Abstract

**Figure:**
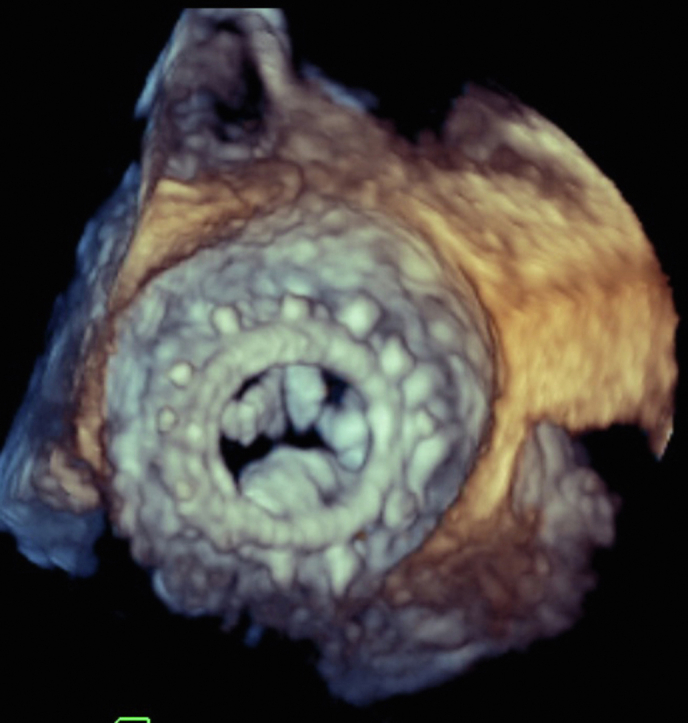

## History of Presentation

**Figure 1:**
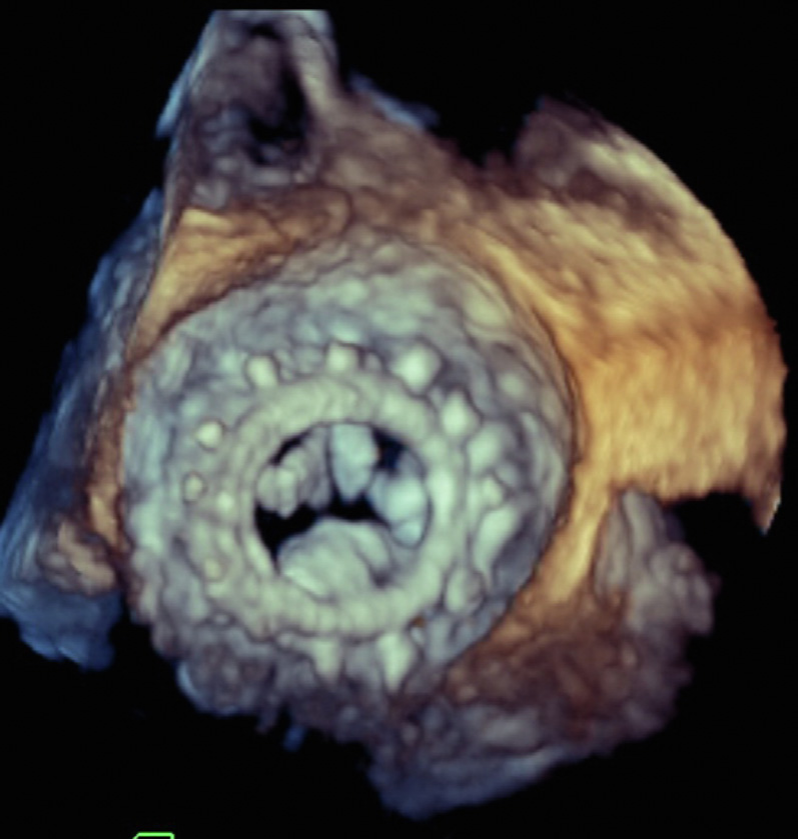
Prosthetic Mitral Stenosis A 3-dimensional transesophageal echocardiogram demonstrating thickened prosthetic mitral valve leaflets. The mitral valve area by planimetry was measured to be 1 cm^2^.

A 78-year-old woman with a history of bioprosthetic mitral valve replacement was admitted with acute congestive heart failure. Her vital signs were blood pressure of 110/70 mm Hg, heart rate of 70 beats/min, and oxygen saturation of 95% on room air; however, her jugular venous pulses were +2, bilateral basilar crackles were present on posterior chest wall, and +2 pitting edema was present in the bilateral lower extremities. Her transesophageal echocardiogram showed signs of bioprosthetic mitral valve degeneration with severe stenosis (Figure 1). She was optimized medically, and after being deemed a poor surgical candidate for redo valve surgery, she was worked up and scheduled for transcatheter mitral valve replacement (TMVR) with a 26-mm balloon-expandable valve. The patient was paced with a temporary pacing wire in the right ventricle to 180 beats/min, and the new valve was positioned over a stiff guidewire (Video 1). The balloon inflation was set to nominal pressure (Video 2). The patient, however, post–balloon inflation, was notably hypotensive, with a blood pressure of 90/80 mm Hg, heart rate of 120 beats/min, and sinus tachycardia on telemetry. She was immediately started on vasopressors. Transeptal access was closed with a 14-mm occluder device with no hemodynamic improvement.

**Take-Home Messages**

• This case highlights the importance of recognizing left ventricular perforation following ViV interventions and the use of multimodality imaging in identifying this defect.
• It also stresses the relevance of performing these procedures in areas where prompt transfusions and cardiac surgery are readily available.

## Past Medical History

Six years ago, she underwent single-vessel coronary artery bypass grafting (venous graft to diagonal 1), bioprosthetic aortic valve replacement for severe aortic stenosis, and bioprosthetic mitral valve replacement for severe mitral stenosis from significant mitral annular calcification. She also has a history of significant pulmonary hypertension with normal right ventricular function (estimated right ventricular systolic pressure of 70 mm Hg by transthoracic echocardiogram [TTE]).

## Differential Diagnosis

The differential diagnosis for a patient in shock following TMVR included valve embolization following valve-in-valve (ViV) deployment, left ventricular outflow tract obstruction from device placement, cardiogenic shock from cardiac tamponade, transeptal perforation, left ventricular perforation, and hemorrhagic shock from a vascular access site complication.

## Investigations

**Figure 2:**
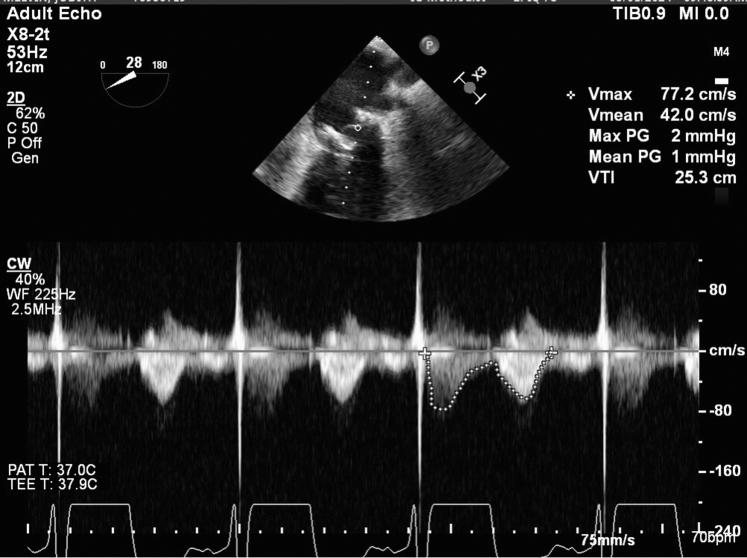
Mitral Valve Gradient Transesophageal echocardiogram showing the mitral valve gradient post–valve-in-valve deployment.

**Figure 3:**
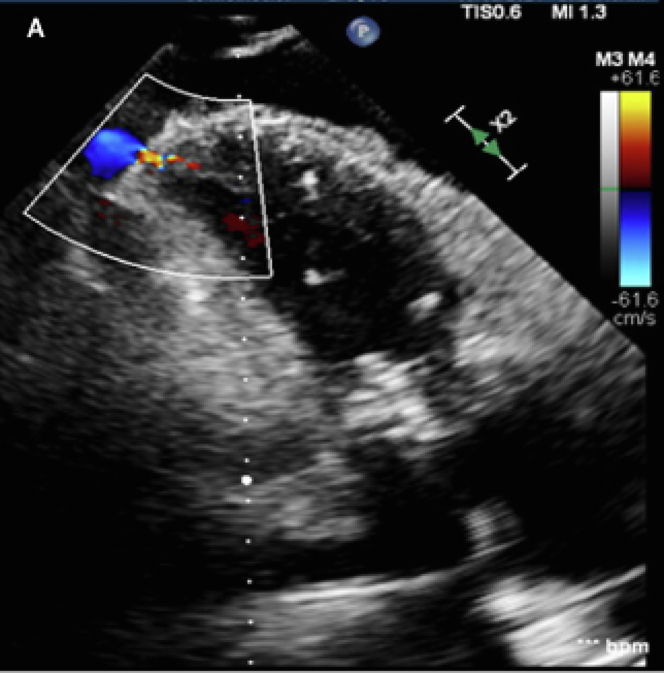
Left Ventricular Perforation Transthoracic echocardiogram with color Doppler showing perforation at the apex of the left ventricle.

The transesophageal echocardiogram postdeployment showed no abnormal motion of the ViV prosthesis (Video 3), and the mean mitral valve gradient was normal (Figure 2). There was a hyperdynamic left ventricle with a large apical pericardial effusion on TTE (Video 4), and color Doppler at the apex showed left ventricular perforation (Figure 3, Video 5).

## Management

**Figure 4:**
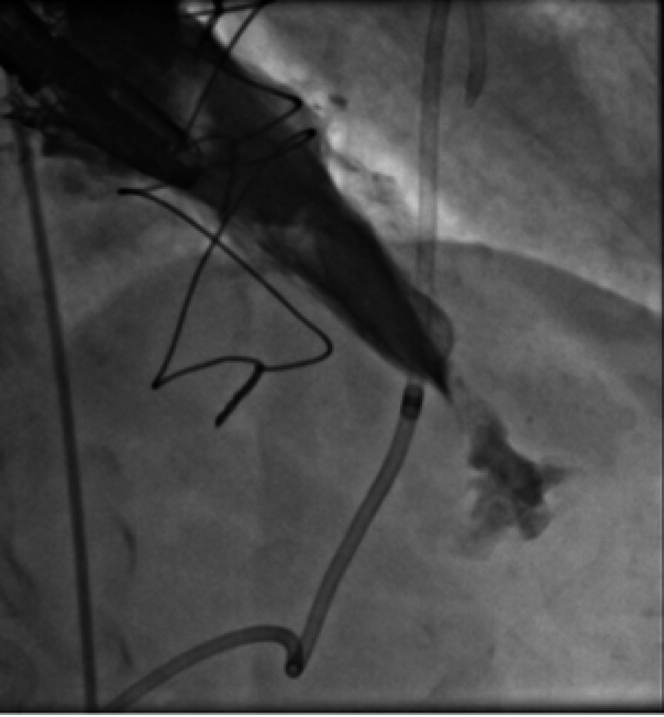
Left Ventricular Perforation Left ventriculogram showing sizable perforation at the apex of the left ventricle.

**Figure 5:**
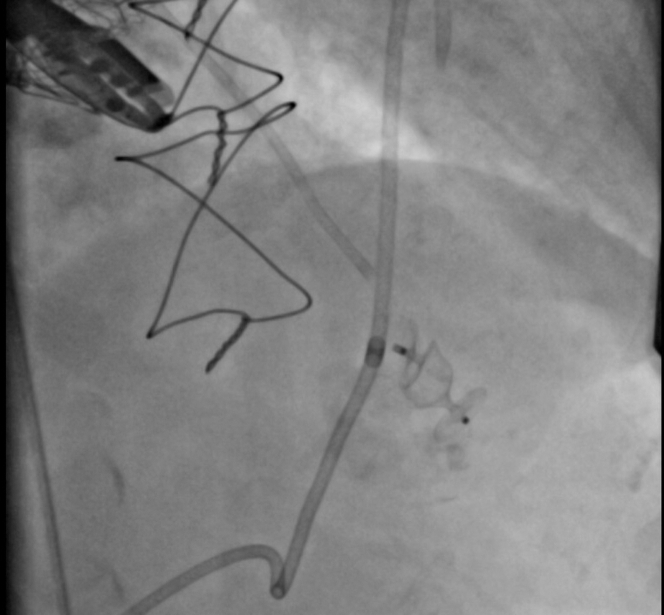
Vascular Plug Deployment of a 12 × 100–mm vascular plug in left ventricular apex.

Patient was urgently given 40 units of protamine. Emergent pericardiocentesis was performed because the size of the left ventricular perforation appeared small by echocardiography, a pericardial drain was placed with brief improvement of blood pressure to 100/80 mm Hg, and the heart rate decreased to 100 beats/min. The bleeding, however, continued, and the pericardial drain filled quickly. We did not autotransfuse the patient with blood from the pericardial drain, and there was a delay in blood products reaching the catheterization laboratory; therefore, the patient did not receive a rapid blood transfusion. We discussed sending our patient to the operating room for emergent apical left ventricular repair. With further hemodynamic compromise, an attempt to plug the left ventricular perforation was planned as a bridge to the operating room. After obtaining arterial access, a multipurpose guide catheter was advanced over a guidewire, selected for the left ventricle, and advanced into the pericardium. A left ventriculogram demonstrated a sizeable extravasation of contrast into the pericardium (Figure 4, Video 6). A 12 × 100–mm vascular plug was advanced through the guide catheter to the left ventricle and successfully deployed into the apex (Figure 5, Video 7). Hemodynamics improved immediately, and vasopressors were able to be weaned down. In the operating room, the perforation site was identified and was still actively bleeding; 2-0 pledgeted sutures were placed around the site of perforation. The site was then observed for several minutes with no evidence of active bleeding from the site.

## Outcome and Follow-Up

After the procedure, the patient recovered smoothly, with repeat TTE on postoperative day 4 showing normal left ventricular systolic function with no residual pericardial effusion. She was also placed on oral anticoagulation to decrease the likelihood of recurrent valvular degeneration.

## Discussion

The only indication for TMVR approved by the U.S. Food and Drug Administration is for ViV interventions for failed bioprosthetic mitral valves using the Sapien Transcatheter Heart Valve System (Edwards Lifesciences) for patients who are at high risk for redo surgical valve replacement. Although a redo operation is typically the standard of care for failed bioprosthetic valves, many of these patients have multiple comorbidities and are older. In patients with a high or prohibitive surgical risk, transcatheter ViV interventions are a Class 2a indication (Level of Evidence: B), as per the 2020 valve guidelines.^1^ This procedure is becoming more common, and according to the Society of Thoracic Surgeons/American College of Cardiology Transcatheter Valve Therapy Registry, 3,597 patients underwent TMVR between 2014 and March 31, 2020.^2^ Within that time, annual procedure volumes for TMVR have increased from 84 per year in 2014 to 1,120 per year in 2019 at 301 centers. In the largest observational study to date for TMVR ViV using the Sapien valve, the success rate for 1,529 patients with degenerated bioprosthetic mitral valves was nearly 97%, and all-cause mortality at 30 days was near 5% and was 16.7% at 1 year.^3^ TMVR ViV also led to clinically meaningful improvements in NYHA functional class III to IV symptoms at 1 year compared to baseline (9.7% vs 87.1%).

Some of the challenges involved in transcatheter therapies for ViV interventions include complex anatomy, patient selection, and proximity of the left ventricular outflow tract. Key portions of the procedure include recognizing complications such as valve embolization and left ventricular perforation. Valve selection is based on the true internal diameter of the degenerated prosthetic valve, and this can be obtained from valve manufacturers or the ViV Mitral application. This careful planning helps reduce the risk of valve embolization. It is vital to recognize left ventricular perforation early, and it can happen at any stage of the procedure, including during manipulation of the guidewire for placement of the valve, during balloon inflation, and after valve deployment. We hypothesize that in our case, the introduction of the stiff guidewire in combination with our patient’s hyperdynamic left ventricle may have led to straightening of the distal end of the guidewire and, ultimately, ventricular perforation. It is unclear when this may have happened during the procedure but was immediately recognized with hemodynamic instability following valve deployment and quickly identified with echocardiographic imaging. Similar case reports have also been described in the literature.^4^

Overall, left ventricular perforation, with a rate <1% in most studies, is an infrequent, but fatal complication of mitral valve interventions.^5^ Similar cardiac perforation injuries also have the potential to occur during transcatheter aortic valve replacement procedures and have been noted to occur in 1.3% of patients in the CoreValve trial. There are only a handful of reports in the literature that address patient outcomes after left ventricular injury during percutaneous valve replacement, and as may be expected, they outline the rapid development of cardiogenic shock requiring open surgical exploration or repair and result in dismal outcomes, with an average mortality of 75%.^6^ In our case of left ventricular perforation, the vascular plug was a temporizing measure that slowed but did not resolve the leak. Moving the patient from the catheterization laboratory to the operating room will often take nearly an hour, thus necessitating a temporizing maneuver. Performing TMVR cases in a hybrid operating room may be one way of reducing this delay, as is frequently done for transcatheter aortic valve replacement, and should be the standard for transcatheter valve procedures. Although we were able to palliate this complication in the catheterization laboratory, it is important that this procedure be performed in areas where prompt transfusions and cardiac surgery can be achieved.

## Conclusions

A 78-year-old woman underwent TMVR for severe bioprosthetic mitral valve stenosis, and this was complicated by shock from cardiac tamponade and left ventricular perforation. This case provides important insights into how to recognize this complication early and offers palliative steps that can be taken before going to the operating room.

## Funding Support and Author Disclosures

The authors have reported that they have no relationships relevant to the contents of this paper to disclose.

## Appendix

Video 1 Valve Positioning Cineradiography showing positioning of the new valve in valve.

Video 2 Balloon Deployment Cineradiography showing deployment of the balloon to nominal pressure.

Video 3 Post–Valve Deployment Transesophageal echocardiogram post–valve deployment.

Video 4 Post–Valve Deployment Transthoracic echocardiogram post–valve deployment.

Video 5 Left Ventricle Perforation Transthoracic echocardiogram with color Doppler showing perforation at the apex of the left ventricle.

Video 6 Left Ventriculogram Left ventriculogram demonstrating extravasation of contrast into the pericardium.

Video 7 Vascular Plug Vascular plug in left ventricular apex showing less extravasation of contrast during contrast injection of the left ventriculogram.
